# Views, barriers, and facilitators of people living with human immunodeficiency virus and healthcare professionals regarding the use of a mobile health application to improve HIV self-care in Malaysia

**DOI:** 10.1371/journal.pone.0349144

**Published:** 2026-05-22

**Authors:** Wen Wei Chung, Pauline Siew Mei Lai, Sasheela Sri La Sri Ponnampalavanar, Thiam Kian Chiew

**Affiliations:** 1 Department of Pharmacy, Universiti Malaya Medical Centre, Kuala Lumpur, Malaysia; 2 Department of Primary Care Medicine, Faculty of Medicine, Universiti Malaya, Kuala Lumpur, Malaysia; 3 Department of Medicine, Faculty of Medicine, Universiti Malaya, Kuala Lumpur, Malaysia; 4 Department of Software Engineering, Faculty of Computer Science and Information Technology, Universiti Malaya, Kuala Lumpur, Malaysia; Touro University California College of Pharmacy, UNITED STATES OF AMERICA

## Abstract

**Aim:**

The views, barriers, and facilitators of people living with HIV (PLWH) and healthcare professionals (HCPs), regarding the use of a mobile health application (m-HA) to improve HIV self-care were explored.

**Subject and Methods:**

Seventeen PLWH, nine doctors and two nurses who owned a smartphone and understood English or Malay were recruited from the infectious disease clinic of a tertiary hospital in Malaysia. Semi-structured in-depth interviews were conducted face-to-face using a topic guide, audio-recorded and transcribed verbatim. Data was analysed using reflexive thematic analysis. Two researchers independently coded transcripts and met to resolve discrepancies and develop themes. Data collection continued until thematic saturation was achieved.

**Results:**

Three themes emerged: (1) Perceived benefits and concerns regarding m-HA usage: participants perceived m-HA could improve knowledge, but expressed concerns regarding information overload. Participants viewed m-HA as a means to enhance access to hospital services like clinic visits, medication refills, communication with HCPs, and medical records documentation. However, concerns regarding misuse, data confidentiality and security remained. (2) Perceived motivators of m-HA: participants perceived that high utilitarian and hedonic motivators increase m-HA usage. (3) Perceived influences to use m-HA: participants believed PLWH would use m-HA if introduced by their peers or HCPs.

**Conclusion:**

These findings provided information for developing a patient-centred m-HA to improve HIV self-care in Malaysia.

## Introduction

Human immunodeficiency virus (HIV) self-care (defined as assuming responsibility to care and manage one’s disease) plays a pivotal role in improving the health of people living with HIV (PLWH) [1]. While HIV self-care broadly refers to the daily behaviours undertaken to maintain health, self-management explicitly describes the skills required by PLWH to manage HIV. In this study, we conceptualised HIV self-care as a broader concept that incorporates both routine health-maintenance behaviours (adherence to antiretroviral therapy and clinic attendance) and self-management skills required for PLWH to address ongoing medical and psychosocial challenges (problem-solving, decision making and collaboration with healthcare providers) while living with HIV.

Collectively, effective HIV self-care support physical, psychological, and social well-being and enable PLWH to take an active role in healthcare decision making, thereby, potentially improving health outcomes and care efficiency. However, living with HIV can be burdensome, as PLWH often face ongoing medical challenges alongside psychosocial vulnerabilities that hinders engagement in self-care behaviours, such as access to medical services, engagement in physical activity and adherence to antiretroviral therapy (ART) [1].

Effective HIV self-care skills include adhering to medications, problem solving, decision making, resource utilisation, formation of a patient-provider partnership, action planning, and behaviour change (setting goals and self-monitoring). This also includes patients’ tailoring management (managing negative emotions, adapting to the nature of the illness, coping with HIV-related conditions, and developing positive social and family relationships) [2]. Hence, effective HIV self-care may improve the physiological, psychological, and social functioning of PLWH and enable PLWH to take an active role in healthcare decision-making. This, in turn, may translate to better healthcare outcomes, thereby reducing healthcare costs and improving the efficiency and quality of care given [3].

Common and effective strategies that may improve HIV self-care skills include counselling on how to live with HIV, training and provision of symptom management and methods to improve medication adherence [2]. Disclosure of HIV status and support from healthcare providers are also pivotal aspects to improve HIV self-care [2]. These key elements initiate support and decrease fear of discrimination, which then improves HIV self-efficacy and self-care [2].

Mobile health applications (m-HA) provide a platform that complements the medical care administered to PLWH [4]. Existing literature suggests that PLWH generally perceive m-HA intervention positively as they enhance opportunities for HIV self-care through the utilisation of innovation and technology. m-HA provide PLWH with convenience, privacy, and potential to reduce stigma-related barriers to care, thereby facilitating greater engagement in HIV self-care [1]. However, studies also report concerns regarding confidentiality, digital literacy, personalization, and cultural relevance especially in low resource settings. These findings highlight the need for a m-HA that are not only technologically functional but also contextually responsive to users’ psychosocial and structural realities.

Several digital interventions have been developed to support PLWH. In China, the Golden Ark application was effective in reducing HIV transmission risk behaviours [5]. Similarly, in Canada, the WelTel application utilized a text-based communication to enhance medication adherence, thereby effectively reduced viral loads in PLWH [6]. In the United States of America, the VIP-HANA application enabled PLWH to monitor their symptoms and communicate with their healthcare providers [7], and in Indonesia, the RumahSELA application was effective in improving HIV-related knowledge, behaviour outcomes, and access to HIV services [8].

These interventions demonstrated feasibility, acceptability and measurable clinical or behavioural benefits, each targeting specific aspects of HIV self-care rather than integrating comprehensive HIV self-care components within a single m-HA. These studies also focused on the use of mobile or web-based interventions (short text messages, advertisements) or web-based advertisements, instead of a m-HA. To date, no m-HA with integrated features required to improve self-care in PLWH has been developed for the non-homogeneous PLWH population, characterised by diverse backgrounds and complex social determinants, in lower-middle-income countries such as Malaysia. This suggests a gap in the development of a holistic, multi-component m-HA platform designed to address the complex and multidimensional HIV self-care needs of PLWH. However, before developing a m-HA, we need to understand the views, perceived barriers, and facilitators of using m-HA to improve self-care in PLWH in Malaysia. Thus, we aimed to explore the views, barriers, and facilitators of PLWH, doctors, and nurses, regarding the use of a m-HA to improve self-care in PLWH in Malaysia.

## Materials and methods

Qualitative in-depth interviews (IDI) were used to explore the views, perceived barriers, and perceived facilitators of PLWH, doctors and nurses in the Infectious Disease (ID) Clinic of a tertiary hospital in Malaysia from 10 January to 6 March 2020. All IDIs were conducted face-to-face before the COVID-19 pandemic.

IDIs were conducted to maintain the anonymity and privacy of PLWH. Hence, IDIs were conducted with eligible participants to determine their preference for content and utility to inform the design of a m-HA aimed at improving HIV self-care in Malaysia. Recruitment of participants ceased once thematic saturation was achieved.

### Ethics approval

Ethics approval was obtained from the Ethics Committee before the study was carried out (approval number: 2019713−7640).

### Conceptual framework

Two theories were used, the Theory of Planned Behaviour (TPB) and the revised Unified Theory of Acceptance and Use of Technology (UTAUT 2) (S1 Fig.) [9,10]. The TPB was selected as it predicts PLWH’s intention to engage in the use of the m-HA. It is posited that behavioural intention and achievement is determined by both motivation (intention) and ability (behaviour control). TPB comprises of three aspects: 1) attitude towards behaviour, 2) perceived behavioural control [9] and 3) subjective norm, which aligns with the concept of self-care [9]. In this study, TPB informed the development of data collection by guiding the inclusion of constructs related to behavioural beliefs, social influences, and control beliefs. These constructs also shaped the analytical framework and interpretation of findings, particularly in PLWHs’ motivation and ability to adopt the m-HA.

The UTAUT 2 model enables researchers to comprehend user acceptance and rejection of utilitarian and hedonic motivators of a m-HA. Utilitarian motivators are functional, assisting users to perform tasks efficiently and objectively focusing on perceived ease of use and usefulness of the m-HA [11,12]. Alternatively, hedonic motivators are emotional, reflecting users’ subjective experiences of enjoyment, excitement and self-fulfilment from using the m-HA [11,12]. UTAUT2, guided both data collection and analysis by identifying key determinants of technology acceptance including performance expectancy, effort expectancy, social influence, facilitating conditions, hedonic motivation, price value, and habit. These constructs were incorporated into the study instruments and subsequently used to structure the thematic analysis and interpretation of user acceptance and rejection of the m-HA’s utilitarian and hedonic features.

The integration of TPB and UTAUT 2 provides a comprehensive framework for understanding PLWH’s intention to use m-HA to improve self-care (S1 Fig.). Both models consider background factors (demographic characteristics, social influence, personal and cognitive factors) that shape PLWHs’ attitude (behavioural beliefs), perceived behavioural control (control beliefs and hedonic motivation), as well as subjective norms (peer and external influences) towards their intention to use m-HA.

### Instruments used

Two instruments were used: a baseline demographic form and a topic guide (Table 1) developed based on literature and the experience of the research team.

**Table 1:** Topic guide used for people living with HIV and healthcare professionals.

TOPIC GUIDE
1. What do you understand by the term mobile health application “m-HA”? What are your experiences using m-HA in managing your health / in your practice? 2. What are your thoughts on using m-HA to assist in managing your health condition / in your practice and to counsel or educate people living with HIV? 3. What are the benefits of using a m-HA? 4. What are your views of using m-HA to provide information, assess adherence or contact a healthcare professional? 5. What encourages you to use a m-HA to manage your condition / in managing people living with HIV / recommending to people living with HIV? 6. What prevents you from using m-HA in managing your condition / in people living with HIV / in recommending to patients?

### Participants

#### People living with human immunodeficiency virus.

PLWH aged 21–64 years, diagnosed with HIV [defined as having a CD4 count (T-cell count) of >200cells/mm3] and receiving treatment in a tertiary hospital in Malaysia for at least 3 months, who own a smartphone, and could speak English or Malay, were recruited. This age group was selected as it represents the majority of the PLWH in Malaysia. PLWH >65 years old (“baby boomers”) were excluded as only <9% of PLWH in Malaysia are ≥ 50 years of age [13,14]. In addition, older PLWH do not respond well to ART, have more co-morbidities [15] and only 3.9% are capable of using a m-HA [16].

Hence, PLWH were purposively sampled based on age. Younger participants aged 20–65 years (generation X, Y, and Z) were recruited. PLWH who had a cognitive or psychiatric disorder were excluded.

#### Doctors treating people living with human immunodeficiency virus.

Doctors treating PLWH, who owned a smartphone and could speak English or Malay, were recruited. Doctors who had less than 6 months of working experience treating PLWH were excluded. Only 6 specialists and 4 registrars fit these criteria. Hence, all doctors were recruited.

#### Nurses providing counselling and education to people living with human immunodeficiency virus.

Nurses who counselled PLWH, owned a smartphone and could speak English or Malay were recruited. Only 2 nurses fit this criterion. Hence, both nurses were recruited.

### Recruitment process

PLWH were identified by screening through their lab results and the type of medications prescribed. For PLWH who fulfilled the inclusion criteria, the purpose of the study was explained to them using the participant information sheet in a consultation room. For those who agreed, written informed consent was obtained, and the interview was conducted immediately, at a private location.

Healthcare professionals (HCPs) were approached in the clinic, and the purpose of the study was explained to them. For those who agreed, their mobile numbers were obtained to set a date or time for an interview.

All participants were asked to fill out the demographic form before the IDI. All IDIs were conducted in a consultation room. Interviews were audio-recorded and lasted for approximately 30–60 minutes. After the interview, initial impressions and thoughts about the participants were recorded in the researcher’s diary. Any additional information post-interview was also included as field notes (S2 Fig.).

### Data analysis

The study adopted the descriptive phenomenology approach as a guiding orientation to explore participants lived experiences from their own perspectives regarding the use of a m-HA to improve HIV self-care. While descriptive phenomenology informed the overall study design and interpretation, data were analysed using Braun and Clark’s thematic analysis to systematically identify patterns and meanings within the dataset [17]. All interviews were audio recorded, transcribed, and translated verbatim. Data analysis followed the following approach: (1) familiarisation with data, (2) generation of initial codes, (3) searching for themes, (4) reviewing themes, (5) defining and naming themes and (6) producing the report [17]. NVivo 11 (QSR International Pty. Ltd. Version 11, 2015) was used to code the themes that emerged. TPB and UTAUT 2 framework informed the analysis at the interpretation stage by guiding the organization and refinement of themes. Coding was conducted inductively to allow themes to emerge from data while the framework was used deductively to map and interpret findings within broader theoretical structure of HIV self-care.

### Trustworthiness

Trustworthiness was established in accordance with qualitative research criteria of credibility, dependability, confirmability, and transferability. All interviews were conducted by WWC (primary researcher), who has more than 10 years of experience as a hospital pharmacist in the same tertiary hospital where the study was carried out. WWC’s positionality was acknowledged and steps were taken to minimize potential bias or role-based influence. Reflexivity was maintained throughout the study via regular discussions with PSML (researcher supervisor). Credibility was enhanced through IDIs that allowed PLWH’s to provide detail accounts of their experiences. Prolonged engagement with the data and iterative review of transcripts further strengthened the accuracy of interpretation.

Dependability and confirmability were ensured through a systematic coding process and maintained of an audit trail documenting analytic decisions. WWC coded each interview line-by-line to develop an initial list of codes (open coding). Subsequent interviews were then coded using this list (a process of constant comparison with the initial open codes created), and new themes that emerged were added to the list. Next, open codes were organised and reorganised conceptually into broader categories based on thematic similarities between open codes or “axial coding.” Throughout the coding process, codes were checked by PSML, SP and CTK to ensure consistency, reduce potential researcher bias and consensus of coding. Finally, core categories and subcategories were organised within each conceptual domain and are conceptually connected thereby generating a theoretical representation of relationships among concepts.

Transferability was supported by providing a clear description of the study context, participant characteristics, and data collection procedures to allow readers to assess the applicability of findings to similar settings.

## Results

A total of 28 participants were recruited: PLWH = 17, doctors = 9, nurses = 2. Median duration of PLWH was 3 years (Table 2).

**Table 2:** Demographic data of participants.

PLWH*	Age (Years)	Gender	Ethnicity	Marital status	Level of education	Years living with HIV
P001	56	Male	Malay	Single	University	3
P002	39	Male	Malay	Single	University	5
P003	42	Male	Chinese	Single	University	1
P004	32	Male	Malay	Single	University	6
P005	22	Male	Malay	Single	Secondary School	1
P006	38	Male	Indian	Married	University	3
P007	36	Male	Malay	Married	University	2
P008	27	Female	Malay	Married	University	1
P009	32	Male	Chinese	Single	University	0
P010	52	Female	Chinese	Married	University	5
P011	47	Male	Indian	Married	Secondary School	1
P012	51	Male	Malay	Single	Secondary School	11
P013	56	Male	Malay	Single	Secondary School	4
P014	29	Male	Malay	Married	University	3
P015	27	Male	Malay	Single	University	5
P016	60	Male	Chinese	Single	University	23
P017	32	Male	Chinese	Single	Secondary School	3
Median (Range)	38 (32-60)					3 (0.5–23)
Doctors	**Age (Years)**	**Gender**	**Number of years treating PLWH**			
D001	32	Male	5			
D002	36	Female	5			
D003	34	Female	5			
D004	47	Female	20			
D005	35	Female	9			
D006	33	Female	3			
D007	34	Female	5			
D008	38	Male	12			
D009	35	Male	5			
Median (Range)	35 (32-47)		5 (3–20)			
Nurse	**Age (Years)**	**Gender**	**Number of years counselling PLWH**			
N001	41	Female	7			
N002	40	Female	7			
Median (Range)	40.5 (40-41)		7 (7)			

* PLWH = people living with HIV

Three themes emerged: (1) perceived benefits and concerns regarding m-HA usage; (2) perceived motivators to use m-HA; (3) perceived influences to use m-HA to improve self-care. Additional illustrative quotations and supporting qualitative data are provided in S1 File.

### Perceived benefits and concerns of using a m-health application to improve human immunodeficiency virus self-care

#### Knowledge regarding human immunodeficiency virus.

Participants believed that a m-HA could improve the knowledge regarding HIV and its treatment for PLWH. They believed that the information provided in a m-HA would be from a reliable source that could provide them with localised information, relevant to their needs. In addition, participants wanted the right information to be provided at the right time, as newly diagnosed PLWH have different needs compared to those with established HIV [18–20]. Upon diagnosis, PLWH need clear and concise information about HIV. As their treatment progresses, they should receive information about routine health monitoring, maintaining a healthy lifestyle, and preventing opportunistic infections. Eventually, comprehensive guidance on long-term management of HIV and new treatment options should be provided [18,19]. This approach would prevent PLWH from feeling overwhelmed by too much information and supports better health outcomes.

*“…resources like AIDS map (a website on HIV/AIDS) delivers reliable information… to healthcare professionals and lay people…” (D004)*

*“…the appeal is towards the newly diagnosis patients as they have a lot more to manage and adjust to…” (P017)*

*“...the other problem is too much information...” (P004)*

There were also requests for features to check for interactions between ART and supplements or over-the-counter medications to assist PLWH in the management of HIV.

*“…record interaction of antiretroviral with supplements and over-the-counter medications…” (P004)*

#### Access to hospital services.

PLWH wanted a m-HA to improve their access to hospital services such as clinic visits, medication refills, and communication with their healthcare provider.

#### Clinic visits.

Most PLWH wanted to schedule their clinic appointment, perform online check-in, and pay online on their m-HA to minimise waiting time and public stigmatisation. In addition, they also wanted the m-HA to remind them when their clinic appointments were.

*“…ability to confirm appointment and pay online can ease clinic visits…” (P001)*

*“Calling in the hospital is a nightmare; it is difficult to get through and even if you do there’s hesitancy to share information, fearing you may get transferred to someone else… it’s frustrating.” (P010)*

Besides, HCPs also suggested a treatment checklist for the monitoring of the recommended vaccinations for PLWH.

*“…a treatment checklist, vaccination reminders... reminds doctor and patient which tests are due...” (D003)*

#### Medications.

Additionally, PLWH also wanted the m-HA to be able to check the availability of their medication at the pharmacy and to remind them when to collect their medications.

*“The features to … check the availability of the medication, medication reminders…will help...” (P001)*

#### Ability to communicate with a healthcare professional.

Additionally, PLWH wanted to communicate with their healthcare provider more efficiently when experiencing side effects, especially when travelling. However, nurses were uncertain about implementing a 24-hour “chat function” as they had concerns about potential misuse and the possibility of medical-legal issues arising when enquiries go unanswered.

*“The feature to …speak to the specialist during an emergency, to check medication availability, schedule appointments…will help...” (P001)*

*“I am struggling as I am not a dedicated nurse for the infectious disease clinic... if we do not answer, patients will get angry…. we are afraid they might misuse the service.” (N001)*

#### Documentation.

There were also requests for the m-HA to monitor their medication adherence and record the side effects that they were experiencing. Additionally, some participants wanted the m-HA to document their “feelings” (like a patient diary) to enable them to manage their health more effectively.

*“…application tracks the medication name, dosage, starting date, a missed medication, a side effect…has a diary and reminder function…can assist us to look after our health.” (P016)*

Participants believed a m-HA could help document and organise their medical information and lab results for PLWH. They believed that a m-HA would make their information more accessible to enable them to monitor their progress better, as improvements seen could motivate them to do better. Nonetheless, doctors believed that PLWH should have personalised goals, as setting targeted goals can assist PLWH to manage their condition more effectively.

*“...good documentation empowers patients to better manage their health, adhere to appointments, look out for side effects, and adhere to medications which improves viral load…this sets a target for them to achieve.” (D003)*

Most participants were also worried about data confidentiality when documenting their medical data in a m-HA.

*“...confidentiality when recording HIV data, is definitely a concern, we’re worried about data leak...” (D007)*

However, a m-HA may not be suitable for everyone, particularly those with poor cognitive function due to neurological manifestations or those who have their own established coping mechanism.

*“I am concerned about individuals with toxoplasmosis (neurological manifestation) …leading to poor cognitive function during that period.” (D001)*

*“…all result can be stored in the hospital system; there is no need to keep such data on my phone... it’s too advance for me…” (P005)*

## Perceived motivating factors to use m-health application to improve HIV self-care

### Utilitarian motivation

#### Perceived security of the application.

Participants were especially concerned about data security due to the stigma and discrimination associated with HIV. Participants would only use the application if it were password protected, had encrypted information, discreet notifications, and avoided the explicit reference to HIV.

*“… patients are fearful of the stigma and discrimination from public…” (P016)*

*“...a secure system and infrastructure can give assurance and improve uptake of the application.” (D009)*

*“…the name is not linked to HIV; the icon is a scenery picture and does not have a red ribbon (mark for HIV) …” (P016)*

#### Perceived ease of use (secure, simple, free and ability to backup).

Additionally, the m-HA should be easy to use and “instinctively driven.” It should also be accessible offline, as internet accessibility could vary from one area to another. PLWH would use the m-HA if it were provided free and contained no advertisements. Moreover, PLWH wanted a m-HA that could be backed up when they changed or lost their devices.

***“*** *…application is friendly, simple and no advertisement… provided free….” (P010)*

*“…application can be backed up …when I switch or lose my phone...” (P016)*

#### Language and tone of the application.

Participants wanted the m-HA to be available in both English and Malay. They wanted a m-HA that was non-judgmental and avoided the use of medical jargon.

*“…application with multiple languages, i.e., Bahasa Malaysia, Chinese, Tamil and English, simple, no medical jargons...” (D007)*

### Hedonic motivation

#### Gamification and incentives.

Additionally, participants emphasised the importance of tailoring a m-HA to cater to different age groups. PLWH of a younger generation may have a higher degree of technology proficiency, where gamification would help them engage with the application better, as the approach to grasp attention and communicate information is different.

*“...simple, easy to understand information suited for a variety of education levels… pictures or illustrations to help those who are illiterate to comprehend the content… “(D007)*

*“…getting information across the millennial generation is challenging …a creative way is required to grasp their attention.” (D003)*

Participants also requested a m-HA that could provide reward or incentive programs to engage and continue the use of m-HA.

*“…to qualify for a 10% discount on the insurance premium, we need to input our health data and achieve specific target levels. This serves as an incentive to use the application and monitor our health.” (P001)*

#### Perceived influence to use m-health application to improve HIV self-care.

PLWH said that they were more likely to use m-HA if it was introduced to them by their peers or by their HCPs. While HCPs felt that the usage of a m-HA would improve if it were incorporated as part of PLWH’s treatment plan.

*“… if application is introduced to me by my friends in KLASS (an NGO society in Malaysia that assists PLWH), I will use it …can help me connect and share with others in the same situation…” (P015)*

*“...if it was recommended to me by my doctor and he thinks that it can help me …of course that would encourage me to use it.” (P004)*

*“…best way to encourage patients to use the application is to provide them with incentives or incorporate the application as part of their treatment plan...” (D003)*

## Discussion

Most participants believed that a m-HA could improve HIV self-care among PLWH, such as providing convenient access to health information, medication reminders, and support networks. PLWH perceived m-HA as a tool to manage their health, enhance health literacy and facilitate communication with their HCP. However, concerns were raised about data privacy, security, and risk of misuse, given the stigma around HIV. Participants were also worried about accessibility, particularly for those in rural or low-resource areas.

Knowledge of HIV self-care education is pivotal in empowering PLWH to take charge of their HIV self-care routines. However, key concepts of HIV self-care education should be localized and communicated in manageable chunks, as inundating PLWH with too much detail can lead to overwhelm and hinder effective HIV self-care [3,21–23]. Our findings were similar to previous studies, which reported that for health education to be effective, the information provided should be from a trusted or reliable source, constantly updated, able to cater to different types of individuals, avoid overwhelming content, and presented in an easily understandable manner [3,21–23]. The ability of the application to improve HIV-related knowledge further reinforces the role of performance expectancy in the adoption of a m-HA, as PLWH perceived m-HA is a useful tool for enhancing HIV self-care. From the perspective of TPB, increased knowledge may strengthen positive behavioural believes, thereby promote favourable attitudes toward the use of m-HA to improve HIV self-care.

In addition to HIV self-care education, facilitating clinic outpatient visits was also crucial for effective HIV self-care. HCPs can reinforce key concepts in HIV self-care education, clarify doubts, and individualise treatment plans for PLWH during clinic visits. However, studies identified that PLWH missed their appointments due to cost, transport, waiting time, stigma, family pressures, busy work schedules or work commitments, forgetting appointment dates, long waiting hours in the clinic, and poor patient-doctor relationships [24,25]. Delayed linkage to care, missed appointments, and poor retention in care has been associated with increased morbidity and mortality in PLWH and risk of new infections [26].

Features in a m-HA, such as a treatment checklist, appointment reminders, and medication alerts, facilitate a structured environment that offers consistent support and guidance to PLWH throughout their outpatient clinic journey. Similarly, previous research on m-HA appointment systems also reported positive outcomes in reducing no-show rates, improving clinic waiting times, which ultimately enhances patient satisfaction and retention in care [22,24,25]. Effective ART requires a 95% adherence to ART regimen [20]. Studies highlight significant improvements in adherence to ART and a decrease in viral load counts when utilising m-HA reminder features [1]. The paucity in HIV knowledge or ART has been linked to poor medication adherence and improper consumption of the antiretroviral regimen, leading to treatment failure and viral resistance [27]. Therefore, equipping PLWH with knowledge and information of their condition is crucial to support their self-care decisions when engaging with HIV self-care.

Communication with HCP was also important to our participants. The ability to communicate with a HCP fosters a patient-provider partnership, as PLWH can discuss sensitive issues and engage in shared decision-making, thereby improving HIV self-care skills [20,22,23,25]. This was consistent with findings from previous studies suggesting that a robust patient-provider partnership, coupled with high-quality communication, kindles an increase in patient engagement in HIV self-care and adherence to ART [1,22,23]. This reduces the perceived stigma of HIV and barriers to healthcare, thereby improving clinical outcomes and retention in care for PLWH [25,26]. The features of a m-HA that facilitates clinic visits, reminders and communication with healthcare professionals reflects the importance of facilitating conditions which is a key construct within UTAUT 2. When PLWH perceive that adequate resources and support are available, PLWH were more likely to adopt new technologies. This is similar within the TPB where these features enhance perceived behavioural control, as PLWH felt more capable in managing their HIV self-care using a m-HA.

The goal of ART is viral suppression. This can be achieved through monitoring of treatment adherence and tracking of HIV progression via the documentation of medical and laboratory records [20]. The documentation of medical and laboratory records in an m-HA facilitates PLWH to engage in meaningful discussions with HCPs, leading to better management of their condition [22,23,28]. The ability for PLWH to digitally document and track changes over time may facilitate a more active engagement in HIV self-care by enabling users to monitor their progress. Such functionality reflects performance expectancy within the UTAUT 2 as PLWH perceived that the application could enhance the efficiency of managing personal health information. In the same time, access to organized medical data may strengthen PLWHs’ perceived behavioural control, a key construct of TPB by empowering PLWH to take on an active role in managing and making informed decisions regarding their health [28,29]. Together, these findings suggest that integrating features that support self-monitoring and record management may promote greater autonomy and engagement in HIV self-care among PLWH. However, targeted and personalised goals should be established to assist PLWH to manage their condition more effectively [20].

Besides, considerations regarding data accuracy, compatibility with existing healthcare systems, user acceptance, accessibility to m-HA (mobile devices and availability of internet) should be considered [22,23]. This aligns with previous studies, emphasizing the importance of setting achievable targets and addressing concerns to enhance HIV self-care skills [3,20,28].

HIV is associated with stigma and discrimination; hence, any m-HA should adhere to the highest standards of security measures [20,23,30]. Studies have shown that more than half of m-HA users disengaged from using an m-HA due to privacy concerns and security breaches [3,20,30]. Our participants wanted higher standards of authentication features to safeguard data confidentiality, security, and privacy of PLWH. This would enhance the trust of PLWH in using m-HA to improve self-care among PLWH [22,30].

The m-HA should be easy to use, “instinctively driven,” and downloaded for free, as an individual’s willingness to use m-HA can be hindered by scepticism and fear of unfamiliar technology [22,28]. The lack of knowledge or guidance to use m-HA contributes to low self-efficacy, particularly among individuals from the baby boomers’ generation, who were identified to have lower technology literacy [21]. Baby boomers are characterized as digital immigrants who were born before the widespread adoption of digital technology [21]. Generation X, Y, and Z are digital natives who are more comfortable with technology and could immerse themselves in new digital tools, adapting to quick m-HA solutions [21]. Although baby boomers have incorporated technology in the communication and entertainment aspect, some are still sceptical and face challenges in navigating complex mobile applications, as effort expectancy constructs such as utilitarian and hedonic motivators influence the time and effort spent in adopting and continuing use of m-HA [3,9,21]. These findings highlight the importance of effort expectancy within the UTAUT 2, which refers to the degree of ease associated with using a technology. When a m-HA is perceived as simple, secure, and accessible, PLWH are more likely to adopt and continue using it. Such design features may also enhance PLWHs’ perceived behavioural control within TPB by reducing technological barriers and increasing confidence in their ability to use the m-HA effectively.

Additionally, our findings also indicate that PLWH desired a m-HA that delivers information regarding HIV in a non-judgmental, professional tone and avoids the use of medical jargon. Studies highlighted the importance of maintaining a balance in m-HA design, ensuring attractiveness, amusement, and non-judgmental communication while preserving a sense of authority and respect [20,21,23]. The heuristic usability model can be adopted during the m-HA development phases to achieve these goals [21,23].

The incorporation of hedonic motivators, including gamification, rewards, and incentives, was found to enhance the utilization of m-HA for HIV self-care. Literature has also shown that health education uptake can be improved by providing information creatively and interactively through illustrations or gamification [1]. Educational games make information easily understood, interesting, and memorable for the receiver [1,23]. Therefore, enhanced disease knowledge and understanding empower patients to openly communicate with HCP and actively contribute to collaborative decisions regarding their HIV treatment and overall care [3,28].

These findings align with the concept of hedonic motivation in the UTAUT 2, which emphasizes the role of enjoyment and intrinsic satisfaction derived from technology use in shaping behavioural intentions. In the context of m-HA, gamified and incentive-based features may complement utilitarian benefits by enhancing user enjoyment, thereby strengthening positive behavioural intentions towards the application [12,23]. These behavioural intentions contribute to sustained engagement and facilitate the development of habitual use, a key determinant of long-term adoption in UTAUT 2. Consequently, incorporating engaging and interactive elements into m-HA design may be a critical strategy for promoting sustained m-HA use and supporting long-term HIV self-care among PLWH.

Despite perceived benefits of m-HA, our participants indicate greater willingness to use m-HA if introduced by peers or HCPs. This supports the UTAUT 2’s hypothesis, emphasizing the significant role of social influence along with performance and effort expectancy in influencing an individual to adopt a m-HA [3,31]. Studies highlighted the impact of social influence on m-HA adoptions, as individuals with chronic illnesses, such as HIV, tend to seek confirmation of behaviour from others in their environment [3,20]. Participants also stressed the necessity for a secure peer support platform in a m-HA. This platform provides emotional reassurance, motivation, and a sense of belonging for PLWH to enhance their HIV self-care skills, thereby achieving health goals [20,32]. Therefore, recommendations from HCPs and organizations carry significant credibility, influencing acceptance, behaviour, and m-HA usage [21,32].

HCPs have suggested the integration of the new m-HA into standard care as a strategy to improve its acceptance. This aligns with studies advocating for the integration of m-HA with the conventional healthcare system as a strategy to improve acceptance and usage while ensuring continuity and retention in care [32,33]. This integration is seen to reduce healthcare costs as PLWH are empowered to do better while enhancing the efficiency and quality of care given to them [3].

### Strengths and limitations

This study’s strength lies in recruiting multiple stakeholders and achieving data saturation for PLWH and doctors. The participation of two nurses reflects the limited direct involvement with patient care in our centre. Additionally, the overrepresentation of male PLWH may introduce bias, potentially impacting the perspectives and experiences of female individuals concerning the use of m-HA to improve HIV self-care. Future studies should adopt a more targeted recruitment strategy to ensure better representation of female PLWH.

## Conclusion

Participants were generally in favour of using a m-HA to improve HIV self-care. Participant perceived that the m-HA could improve their knowledge, would enable them to communicate with their HCP, and assist them in organizing their medical information so that information is readily available at their fingertips. However, participants expressed concerns regarding data confidentiality, security, and privacy, as HIV is associated with stigma and discrimination. Findings from this study could be used to inform the design and development of a m-HA for PLWH in Malaysia to improve HIV self-care.

The findings also demonstrate how constructs from TPB and UTAUT 2 jointly explain the factors influencing the acceptance of m-HA among PLWH. While TPB highlights the role of behavioural beliefs, social norms, and perceived behavioural control in shaping PLWHs’ intentions; UTAUT 2 clarifies technology-related determinants such as performance and effort expectancy, utilitarian, and hedonic motivation. Integrating these theoretical frameworks provides a comprehensive understanding of behavioural and technological influences on the adoption of a m-HA. These insights offer important guidance for the development of user-cantered m-HA interventions that are engaging, secure, and responsive to the needs of PLWH, particularly in supporting sustained HIV self-care in lower-middle-income countries such as Malaysia.

## Supporting information

S1 Fig Conceptual framework based on the theory of planned behaviour (green) and revised theory of acceptance and use of technology 2 (orange). Description of the theoretical constructs and relationships used to develop the study framework, integrating variables from the TPB and UTAUT 2.
(DOCX)

S2 Fig Recruitment process of participants. Description of the participant enrolment process in the study.
(DOCX)

S1 File Additional supporting dataset. Description of supplementary anonymized participant quotations supporting the qualitative findings in the study.
(DOCX)
