# Supplementary material for: Views, barriers, and facilitators of people living with human immunodeficiency virus and healthcare professionals regarding the use of a mobile health application to improve HIV self-care in Malaysia
Source: PLoS One. 2026 May 22;21(5):e0349144. doi: 10.1371/journal.pone.0349144 (PMC13196958; doi:10.1371/journal.pone.0349144)

**Supporting Information 1 Figure**

**Conceptual framework based on the theory of planned behaviour (green) and revised theory of acceptance and use of technology 2 (orange).**


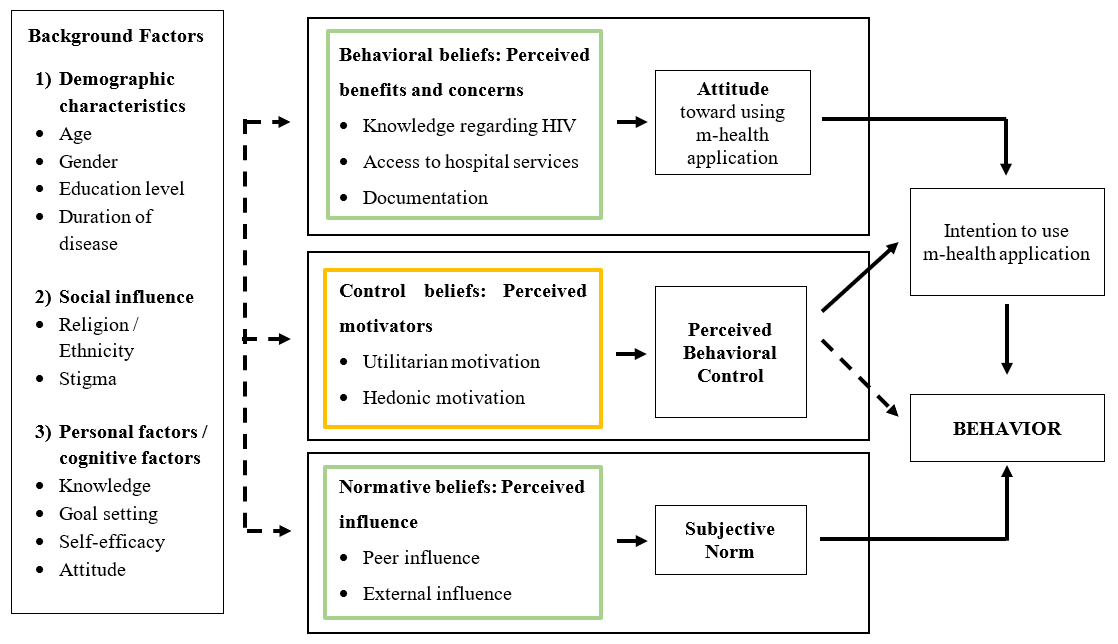

Supplement: S1 Fig — Description of the theoretical constructs and relationships used to develop the study framework, integrating variables from the TPB and UTAUT 2. (DOCX) [file pone.0349144.s001.docx]
