# Supplementary figures and images for: Views, barriers, and facilitators of people living with human immunodeficiency virus and healthcare professionals regarding the use of a mobile health application to improve HIV self-care in Malaysia
Source: PLoS One. 2026 May 22;21(5):e0349144. doi: 10.1371/journal.pone.0349144 (PMC13196958; doi:10.1371/journal.pone.0349144)

**Supporting Information 2 Figure**

**Recruitment process of participants**


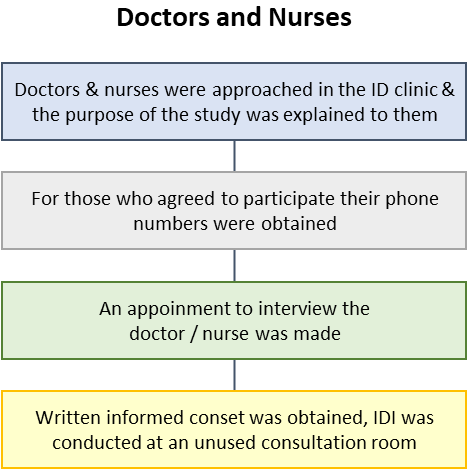

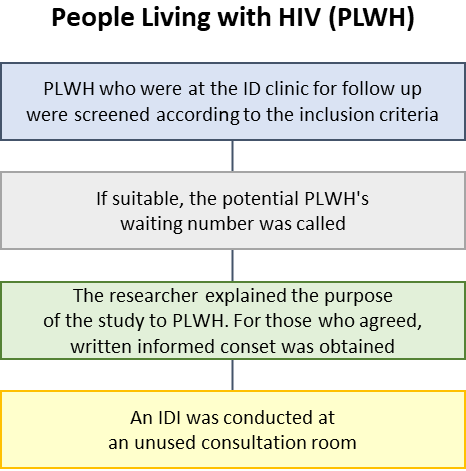

Supplement: S2 Fig — Description of the participant enrolment process in the study. (DOCX) [file pone.0349144.s002.docx]
