# Supplementary material for: Views, barriers, and facilitators of people living with human immunodeficiency virus and healthcare professionals regarding the use of a mobile health application to improve HIV self-care in Malaysia
Source: PLoS One. 2026 May 22;21(5):e0349144. doi: 10.1371/journal.pone.0349144 (PMC13196958; doi:10.1371/journal.pone.0349144)
Supplement: S1 File — Description of supplementary anonymized participant quotations supporting the qualitative findings in the study. (DOCX) [file pone.0349144.s003.docx]

1. **Perceived benefits and concerns of using a m-health application to improve human immunodeficiency virus self-care**
2. **Knowledge regarding human immunodeficiency virus**

- **Knowledge**

D004: …resources like AIDS map (a website on HIV/AIDS) delivers reliable information… to healthcare professionals and lay people…

P004: ...the other problem is too much information...

P012: …a HIV patient would want, the latest news with regards to their medication…we are able to get the latest information on medication… able to get the latest information and determine if the information is reliable or not.

P015: … update about the latest news about the disease…

P017: …the appeal is towards the newly diagnosis patients as they have a lot more to manage and adjust to…”

- **Interaction of medications**

P002: If I know the medication’s name, its drug type, and the substances it contains, I can easily look up how they react with one another online.

P004: …can find and record interaction of antiretroviral (ART) with supplements and over the counter medications…

P005: I would like to see examples of possible side effects included, as well as reminders for appointments and blood tests

P009: …I went online to read in detail about the side effects and interaction of the medications suggested, since what was listed is quite brief.

P012: The most important thing is knowing the medications we take and their side effects…also if they can be taken together

1. **Access to hospital services**

- **Clinic visits**

D003: … doctor reminder visits and blood taking reminders will be helpful, as patients tend to come 2 weeks before their appointment…

D007: reminders would be good for patients… appointment reminders, especially those who have a lot of appointments because sometimes they are seeing 3 to 4 different specialties in a short span of time…

N001: How to get an appointment? Are there services to patients with HIV / STI (sexually transmitted disease)? What is the clinic day? How to make an appointment? Which doctor would I meet? What about payment?

P001: …appointment reminders... ability to confirm appointment and pay on the application to ease clinic visits…

P002: So far, when it comes to appointments, there is no online system. For medical appointments, it is either managed manually using a card or through phone calls. If you are unable to attend a particular appointment, you can inform them by calling

P003: Reminders for appointments, medications, and other health-related matters—such as blood tests or anything concerning my health—could be added into the app

P005: I would like to see examples of possible side effects included, as well as reminders for appointments and blood tests

P008: A reminder system that can be linked to the hospital would be helpful, especially for scheduling blood tests. There could be a section next to the appointment details to ensure that we do not miss appointments or required blood tests.

P010: Calling in the hospital is a nightmare; it is difficult to get through and even if you do there’s hesitancy to share information, fearing you may get transferred to someone else… it is frustrating

P013: The app should provide instructions on how to take medications, is the medication available, how to take care of ourselves, and what we need to do to manage our health. It should also include reminders for follow-up appointments

- **Treatment checklist**

D003: …a treatment checklist, vaccination reminders... reminds doctor and patient which tests are due...

D007: … vaccinations checklist as well or for ladies even yearly pap smears…

D009: …checklist on vaccination and blood test so doctors know the patients have not missed out on vaccines or blood test required when they are taking certain medications.

P003: Yes, it can also help us remember when to take specific vaccines and when to undergo certain blood tests, as well as explain why these are important for our health

P007: Having a schedule would be beneficial because it would allow us to keep track of when vaccinations are due and remind our doctors if necessary…doctors can quickly view the information through the app…serves as a reminder for both patients and doctors.

- **Medications**

P001: The features to … check the availability of the medication, medication reminders…will help...

P003: Reminders for appointments, medications, and other health-related matters—such as blood tests or anything concerning my health—could be added into the app

P005: I would like to see examples of possible side effects included, as well as reminders for appointments and blood tests

P007: … the medication reminder function is useful...

- **Ability to communicate with healthcare professionals**

D001: I am unsure about its feasibility in our setting, as I personally have not used such systems before—apart from simple text messages from patients asking whether their condition is urgent or what they should do… this system could be beneficial because, in the future, it may become one of the easiest ways to disseminate knowledge and reduce the burden on clinics, particularly through virtual consultations.

D002: There should certainly be features that can address patients’ concerns, such as a chat function.

D007: … at current, clinicians already have a lot of issues to address… at the end of the day there’s still only limited number of clinicians with limited amount of time …

D008: …there is going to be quite a lot of investment in time as well as effort from healthcare workers too to cater the needs of patients after office hours…

N001: I am struggling as I am not a dedicated nurse for the infectious disease clinic... if we do not answer, they (patients) will get angry…. we are afraid that they misuse the service.

N002: It makes communication easier and allows us to deliver more effective services… it can reduce our workload... patients may not need to come to the clinic, and healthcare professionals may not need to attend to patients in person. Since the clinic manages not only infectious disease cases but also general cases, this approach can make it easier for patients to seek advice and obtain medical opinions.

P001: The feature to …speak to the specialist during an emergency, to check medication availability, schedule appointments…will help......

P004: When we have just started a treatment, we may tend to overreact, as even a single dose can cause stress and concern that any symptom might indicate something serious. For example, if something appears on the hand, it would be helpful to take a picture and receive some form of guidance—such as whether it is just a mosquito bite, a boil, or something minor. Hence if I can chat with a doctor, it would be very useful.

P008: … I did try to contact the hospital but I guess they (nurses) were busy so they (nurses) did not reply the message… I did not receive any type of information or advice…

P011: Based on the situation, it is helpful to be able to contact a doctor or nurse. For example, when we experience a condition that is unfamiliar or new to us, being able to reach a healthcare provider through a mobile platform would be very beneficial.

P012: We would ask questions because, for instance, we may only see our doctor once every three to four months. This means that any concerns we have might only be addressed much later. If there were a feature within the application, we could ask questions immediately—for example, why we are taking a particular medication or why we are experiencing certain symptoms—and receive feedback on the spot

P014: If I can confirm the possible side effects, I would feel less anxious or worried about new symptoms that I experience. I would also be less likely to rush to the doctor’s office, as I would already have obtained feedback and relevant information through the application.

1. **Documentation**

- **Good documentation**

D007: …app to track medication names, measure their blood pressure and blood sugar will empower patients to take charge of their health…

P007: ...uploading data can help us to keep track of our condition… but to manage, we need to know what we should do to improve our results...

P013: The app should provide instructions on how to take medications, is the medication available, how to take care of ourselves, and what we need to do to manage our health. It should also include reminders for follow-up appointments

P016: …application tracks the medication name, dosage, starting date, a missed medication, a side effect…has a diary and reminder function…can assist us to look after our health

- **Empowerment**

D003: ...good documentation empowers patients to better manage their health, adhere to appointments, look out for side effects, and adhere to medications which improves viral load…this sets a target for them to achieve.

D007: …app to track medication names, measure their blood pressure and blood sugar will empower patients to take charge of their health…

D008: We should move towards empowering the patient towards better care…

P002: …having access to my health records provides greater accessibility and autonomy in tracking my health condition, reducing the need to rely solely on doctors

P006: …I would like to compare my previous blood test readings with the current ones as this would make me more aware of what I have done right or wrong and empower me to improve my health…

P008: …if there is a guidance on what to do with the results it will help me to understand or response to the results, otherwise it will not make much sense to me…

P011: I like to review the results of each blood test. I want to know whether the results are positive or negative, as this provides me with a sense of motivation…

P012: …if I can see that my blood results have improved and understand what that means, it can motivate me and help me remember what I need to do to manage my health

- **Data leak**

D003: …the main concern with the app is security that is really the main concern… most of our patients are very private about their diagnosis

D004: …cyber security is always a concern … using technology to deliver healthcare—is now widely practiced around the world…it is important to weigh the benefits against the risks.

D007: ...confidentiality when recording HIV data, is definitely a concern, we're worried about data leak...

D009: Security is a major concern for any online system. If an individual has the intention to hack, they may attempt to breach the system regardless of the security measures in place.

P001: Of course, any personal information stored should have an appropriate level of security.

P002: …if the hospital can guarantee that there will be no data breach issue, then I don’t see there should be a problem for patients to use that apps…

P012: Another important measure would be to include password protection, so that if someone loses their phone, their information would still not be accessible to others.

P016: Security is very important so that patients can safely access their information.

- **Neurological manifestations / own coping mechanism**

D001: I am concerned about individuals with toxoplasmosis (neurological manifestation) …leading to poor cognitive function during that period

N001: …when a HIV patient is in their toxo phase, they are not 100% in a right mind...

P005: …all that result can be kept just in the hospital, so there is no need to keep such data in my phone... it is too advance for me…

P010: …if the application contains too much information, it could discourage me from using it…I click on one thing and find 50 more options to explore, it becomes overwhelming… I am quite an organized person. I keep a detailed diary, which I refer to frequently—it is almost like a second Bible for me.

1. **Perceived motivating factors to use m-health application to improve HIV self-care**
2. **Utilitarian motivation**

- **Perceived security of the application**

D006: …password protect their phone… app needs a special through password …do not use the specific terms like HIV or viral load... and information should be encrypted….

D009: ...a secure system and infrastructure can give assurance and improve uptake of the application.

P002: it is important to ensure that there is no risk of data breaches… many companies today face issues related to data security including large organizations, like airlines, have experienced security vulnerabilities.

P008: …we will feel more secure if there is a password that we can put in before we open the application…. the icon of the application must be discrete no HIV symbol…

P016: … patients are fearful of the stigma and discrimination from public…the name is not linked to HIV; the icon is a scenery picture and does not have a red ribbon (mark for HIV) …

- **Perceived ease of use (secure, simple, free and ability to backup)**

D001: … the features should be friendly…

D005: …user friendly, simple language and secure…

D009: …the application must be highly intuitive, simple, easy to use and accessible to users with varying levels of digital literacy…

N002: …the application should be simple, segmented, and tailored to different age groups, balancing engagement… gamification for younger users and straightforward, accessible content for older users…

P002: … should be no payment for the application…

P004: …if it cost a lot of money, it would hinder me from using it…

P005: …should use simple, concise, and visually driven content to improve comprehension and enhance understanding….

P010: …if the app is too complicated or has too much information …that will prevent me from using… application is friendly, simple and no advertisement… provided free…

P015: …. application can be upgraded for free or transferred when I switch phones…

P016: …application can be backed up …when I switch or lose my phone...

- **Language and tone of the application**

D004: … language used to provide knowledge and continuous education geared to lay people would be useful…simple language…

D006: … try not to use like medical jargon instead use something common and easily understand words.

D007: …application with multiple languages i.e., Bahasa Malaysia, Chinese, Tamil and English, simple, no medical jargons...

D009: We have of sorts of patients… reads up everything they find on the internet, the ones who are illiterate, the elderly who do not use their mobile phones and the young generation that cannot live without their phones... you need to balance the information in the app to cater for these 4 groups…

P004: …the language should not be harsh....

P014: …. words used should be empathetic and suited to the literacy of the patient...

P016: … the application should be able to switch languages …

1. **Hedonic motivation**

- **Gamification**

D003: …getting information across the millennial generation is challenging …a creative way is required to grasp their attention.

D007: …simple, easy to understand information suited for a variety of education levels… pictures or illustrations to help those who are illiterate to comprehend the content…

D008: …technology is now mature, and users have higher expectations. People are often looking for a unique ‘X factor’ in applications, particularly those with game-like elements… platforms incorporate various strategies to keep users engaged daily. There is a behavioural science behind these engagement mechanisms. If similar principles can be applied, it would enhance user uptake and sustained use.

P009: …presenting HIV information in an engaging way would make it less monotonous and help users better understand their condition…

P015: …instead of putting everything into one long article that they scroll through without reading … a knowledge game would entice them to learn and remember …

- **Rewards and incentives**

D002: …if it comes with some reward for them maybe they are more motivated to do it…when they adhere to their appointments, blood taking and medication maybe they would get some form of reward in terms of free medication or supplements…

D003: …best way to encourage patients to use the app is to provide free things like a complementary gift…a reward for winning the quiz…

P001: …to qualify for a 10% discount on the insurance premium, we need to input our health data and achieve specific target levels. This serves as an incentive to use the application and monitor our health.

P009: …incorporating some form of reward system could be beneficial… without benefits or rewards, it may be difficult to retain users’ interest and encourage continued engagement with the application

1. **Perceived influence to use m-health application to improve HIV self-care**

D003: … best way to encourage patients to use the application is to provide them with incentives or incorporate the application as part of their treatment plan...

D005: …it needs to be user-friendly… we need to understand how to use it ourselves so that we can effectively teach our patients… clear point of contact in case technical issues arise—some form of technical support or backup system is necessary… consider training both healthcare providers and patients to use the application.

D006: If patients specifically inquire about such tools, I would mention them. However, I do not actively promote or impose their use. While I believe it is beneficial to encourage patients to consider these options, I do not view it as appropriate to pressure them into adopting them.

D009: …if I notice that a patient is struggling to remember their medications and I know of an application that could help them—and it is easy to use—then I would recommend that they use it.

P004: ... if it was recommended to me by my doctor and he thinks that it can help me of course that would encourage me to use it …

P015: … if application is introduced to me by my friends in KLASS (an NGO society in Malaysia that assists PLWH), I will use it …can help me connect and share with others in the same situation …

P017: … if the app could connect others who are like me then of course I would use it… When you speak to someone who is experiencing the same condition, it can be more helpful than talking to someone who hasn’t experienced it
